# The etiology of primary femoroacetabular impingement: genetics or acquired deformity?

**DOI:** 10.1093/jhps/hnv046

**Published:** 2015-06-18

**Authors:** Jonathan D. Packer, Marc R. Safran

**Affiliations:** Department of Orthopaedic Surgery, Stanford University, Redwood City, CA 94063, USA

## Abstract

The etiology of primary femoroacetabular impingement (FAI) remains controversial. Both genetic and acquired causes have been postulated and studied. While recent studies suggest that genetic factors may have a role in the development of FAI, there is no conclusive evidence that FAI is transmitted genetically. Currently, the most popular theory for the development of cam-type deformities is that a repetitive injury to the proximal femoral physis occurs during a critical period of development. There is a correlation between a high volume of impact activities during adolescence and the development of cam-type deformities. Multiple studies have found a high prevalence of FAI in elite football, ice hockey, basketball and soccer players. In this article, we review the current literature relating to the etiology of primary FAI.

## INTRODUCTION

**Fig. 1.**
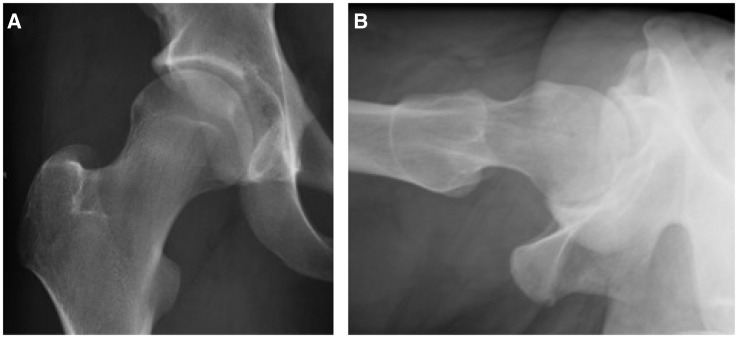
AP (**A**) and cross-table lateral (**B**) radiographs of a young athlete with a prominence at the head–neck junction.

Femoroacetabular impingement (FAI) is the abnormal contact of the proximal femur with the acetabulum. The types of FAI are pincer lesions, cam deformities or both (mixed). A pincer lesion is an acetabular overcoverage (focal or global) whereas a cam deformity is the loss of the normal femoral head sphericity at the head–neck junction (Fig. 1). This aspherical region is typically located anterolaterally on the femoral neck and can lead to damage of the chondrolabral junction. Nötzli originally quantified the cam deformity using the ‘alpha angle’ [1], which is centered in the center of the femoral head and formed by two lines between the femoral neck axis and a line where the femoral head leaves a best-fit circle. While the alpha angle is commonly used to measure cam deformities, the ‘normal’ ranges have not been agreed upon.

The understanding of FAI has revolutionized the treatment of hip pain in the young, non-arthritic patient. While the treatment of FAI continues to evolve [2–4], the etiology of the osseous deformities is poorly understood. Both genetic [5–12] and acquired [13–23] causes of FAI have been postulated and studied in the literature. There is recent evidence to support the concept that the formation of cam lesions occurs with repetitive injury to the proximal femoral physis during a critical period of development [17, 24–29]. Most studies have focused on identifying the cause of the development of cam lesions with few reports on the development of pincer lesions. Further, there is also a lack of theories explaining the etiology of acquired pincers lesions.

### Genetic etiology

Genetic factors have been found to be important in the development of hip osteoarthritis (OA) [30–35]. Some authors have suggested that abnormal joint morphology, such as FAI, may be the underlying genetic predisposition to osteoarthritis. Researchers have been particularly interested in comparing white and Asian populations since low rates of primary OA have been reported in Asians [7, 36–39]. The prevalence of hip OA is 5–10 times higher in white populations compared with Chinese populations of the same age and sex [36, 38]. Asians typically develop hip osteoarthritis as the result of congenital hip disease, such as developmental dysplasia of the hip (DDH), rather than FAI [7, 37]. Takeyama *et al.* [7] retrospectively investigated 946 hips in 843 consecutive Asian patients who underwent primary hip surgery. They found that only 1.2% of hips were diagnosed with primary OA and that only 0.6% of hips had cam deformities (alpha angle >60°). The authors concluded that FAI is rare in Japanese populations.

In a recent cross-sectional study of both white and Chinese asymptomatic individuals, Van Houcke *et al.* [8] concluded that Chinese and white patients differ significantly with regard to hip anatomy. They imaged 201 patients (age 18–40 years) with computed tomography scans and found that the white patients had higher average alpha angles (56°) than Chinese patients (50°). Further, 56% of white patients had an alpha angle >55° compared with 34% of Chinese patients. The average center-edge angle was also greater in white subjects (39°) compared with the Chinese subjects (35°). In a different retrospective study [9] of 87 Japanese patients (80 women) who underwent unilateral hip osteotomy on the contralateral hip, researchers did not find any cam deformities on anterioposterior radiographs. However, there was a crossover sign in 30.6% of hips without dysplasia, which is comparable to the prevalence of acetabular retroversion in western populations [40]. It should be noted that the study populations in these two investigations were all undergoing surgery for a hip condition and are not necessarily representative of the general population.

In a study investigating the role of genetics in the development of FAI, Pollard *et al.* [6] studied 64 FAI patients and compared their siblings (*n* = 96) with a spouse control group (*n* = 77). They found that there is an increased risk of 2.8 for siblings compared with controls of having the same cam-deformity (alpha angle >62.5°) as the patients. The siblings of patients with a pincer deformity had a relative risk of 2.0 of also having a pincer lesion. In the sibling group, 11 of 96 sibling hips had grade 2 osteoarthritis compared with 0 of 77 control hips. When comparing the parents of the study subjects, 13 of the 64 FAI patient parents had osteoarthritis or a hip replacement compared with 6 of the 77 control families. While this study is elegant in an epidemiological approach, it does not conclusively support a genetic link. Certainly there are environmental factors, including athletic activity, that are common within families, especially when raised together. In epidemiologic studies of FAI, it is not possible to separate these social factors from genetic factors.

While these epidemiologic studies were designed to detect differences in the prevalence of FAI and hip OA in different populations, other researchers have reported on the relationship between specific genes and hip morphology. Safran *et al.* [5] investigated the incidence of the single nucleotide polymorphisms (SNPs) at GDF5s rs143383 and Frizzled rs288326 in 69 FAI patients (47 mixed, 16 pincer, 6 cam). These genes were chosen because they are on the developmental locus and have been associated with hip osteoarthritis. GDF5 is involved in development (e.g. chondrogenesis, skeleto-genesis and joint development) and is expressed in the human embryo appendicular skeleton and in adult cartilage. The Frizzled trans-membrane receptor is involved in the pathway stimulating the transcription of cytokines such as IL-6, IL-8 and IL-15 and matrix degradation enzymes such as matrix metalloproteinases. The frequencies of GDF5 and Frizzled in the FAI patients were compared with the general population using the HapMap database and no significant differences were found either in general or when studied by FAI type or gender. When they expanded this study to 100 consecutive patients, the results still did not support either of these SNPs as the etiology of FAI. Although this study did not confirm the two SNP’s are associated with FAI, it does not mean that there is no genetic link to FAI. It may be that other genes that were not studied are potentially involved or that the Hap Map does not accurately represent a general population that was used for comparison.

In a case-controlled study of 1052 Caucasian women (age >65 years), Baker-Lepain *et al.* [10] used statistical shape modeling of hip radiographs to categorize different proximal femur shapes, as well as center-edge angle and acetabular depth. They found that the rs288326 and rs7775 *FRZB* SNPs were associated with a specific shape of the proximal femur. Also, in the presence of the rs288326 variant allele, there was an increased likelihood of developing hip OA associated with this specific proximal femur shape. The authors concluded that *FRZB* may serve an important role in determining hip morphology and may alter the relationship between hip morphology and the development of hip OA.

In another study utilizing statistical shape modeling of radiographs, researchers correlated proximal femur shape with the SNPs of *GDF5, FRZB* and *DIO2* [11]. Four of the 23 hip shapes were strongly associated with OA characteristics. There was a significant correlation between the presence of DI02 rs12885300 and hip OA characteristics for a specific proximal femur shape. These results suggest that this SNP may increase the vulnerability of cartilage to abnormal hip morphology rather than directly influencing the formation of these shapes. The development of OA may be the result of a complex interaction of genes and loading history resulting in abnormal hip morphology and varying abilities of cartilage to withstand mechanical stress (cartilotype) [41]. The concept of cartilotypes may explain why in prospective studies with up to 40 year follow-up, there are a significant number of patients with FAI or DDH who do not develop progressive OA [42, 43].

Sekimoto *et al.* [12] has been the only group to study genetic variation associated with pincer lesions. They investigated the relationship between SNPs of HOX9 genes and acetabular coverage in Japanese individuals. The genotype and allele frequencies of the five HOX9 SNPs had a significant association with acetabular over-coverage compared with controls. The authors concluded that HOXB9 SNPs may be involved in the morphogenesis of acetabular coverage, and could be an independent risk factor for developing pincer-type FAI. Although the results of these studies suggest that genetic factors may have a role in the development of FAI or the susceptibility of hips with FAI to developing OA, future studies are required to better understand this complex relationship.

### Athletes and FAI

There are numerous reports in the literature describing the role of proximal femur deformities and FAI in the etiology of osteoarthritis of the hip [25, 26, 44–49]. In 1971, Murray and Duncan [44] first reported on this association when they found that increased athletic activity in adolescence was a risk factor for the development of hip degenerative joint disease. They found a >3-fold increase of a proximal femoral ‘tilt deformity’ in athletes compared with controls. The authors speculated that excessive activity during adolescence resulted in asymptomatic growth disturbances and a ‘pistol grip’ deformity that eventually predisposed the patient for hip arthritis [44, 50].

In support of these findings, a systematic review found that there was a moderate relationship between sporting activities and the development of hip OA [51]. Other published reports have found that male athletes involved in running and jumping sports have an earlier onset and increased risk of hip OA [44, 52–54]. A retrospective matched cohort comparing former elite athletes with controls found that the athletes had a relative risk of 2.0 of developing hip OA [55]. They also found that athletes of high impact sports were at higher risk of developing hip OA than participants in non-impact sports. In a cross-sectional population-based study (Copenhagen Osteoarthritis Study), Gosvig *et al*. [56] found that pincer and cam deformities were a significant risk factor for the development of OA (risk ratio 2.4 and 2.2 respectively).

There is increasing evidence that participating in high-impact sports during growth plays an important role in the development of a cam deformity [13–18, 21, 22, 27, 57]. This is concerning given the trend toward year-round participation in youth sports with early specialization. Numerous cross-sectional studies of both symptomatic and asymptomatic athletes have demonstrated a high prevalence of cam deformities [14, 15, 18, 22, 27].

In order to identify a group that is at risk for developing FAI, it is important to define the prevalence in the normal population. In studies of asymptomatic individuals, the prevalence of cam deformities has ranged from 9% to 25% in men and 3% to 10% in women [17, 56, 58–63]. In a population-based cross-sectional study, Reichenbach *et al.* [60] obtained magnetic resonance imaging (MRIs) from a random sample of 244 asymptomatic males (mean age 19.9 years). Cam-type deformities were found in 24% of study participants and 91% of lesions were located in the anterosuperior position. Hack *et al.* [59] studied 200 asymptomatic volunteers with an MRI and found that 24.7% of men and 5.4% of women had evidence of a cam deformity (alpha angle >50.5°) in at least one hip. Gosvig *et al*. [56] reported on 1332 male and 2288 female participants in the Copenhagen Osteoarthritis Study. They found that 15.2% of men and 19.6% of women had a pincer abnormality (lateral center-edge angle >45°). The prevalence of cam deformities was 19.6% in men and 5.2% in women. Kienle *et al*. [64] studied 64 subjects (127 hips) from a primary school and a high school to be used as normative values. Baseline and 1-year follow-up MRIs were obtained. The mean alpha angle for all patients was 42.2° ± 8.6° and did not significantly change at 1-year follow-up (42.48° ± 8.79°).

### Prevalence of Cam Deformities in Athletes

#### Football

Recent studies have demonstrated a high prevalence of FAI in collegiate football players [15, 16, 19]. Kapron *et al*. [15] prospectively studied 67 male NCAA Division 1 collegiate football players (134 hips) with radiographs. They found that 95% of hips had at least one sign of cam or pincer impingement and 77% had more than one sign. Seventy-two percent of players had an abnormal alpha angle (>50°), 64% had a decreased femoral head–neck offset and 61% had a positive crossover sign.

These findings were supported by two retrospective studies at the National Football League (NFL) Combine of athletes undergoing hip radiographic imaging [16, 19]. Nepple *et al.* [16] reported on all 107 athletes (123 hips) from 2007 to 2009, who were imaged for a history of hip or groin pain. Cam and/or pincer deformities were present in 94.3% of hips. Mixed-type FAI was the most prevalent (61.8%), followed by isolated pincer (22.8%) and cam deformities (9.8%). A body mass index >35 was associated with the presence of global overcoverage (46.2% versus 17.3%, *P* = 0.025). In a separate study, Larson *et al*. [19] reviewed 132 players (261 hips) undergoing hip radiography at the 2009 and 2010 NFL combines. Ninety percent of players and 87% of hips had at least one finding of cam and/or pincer deformities. There were 75 hips in the symptomatic group and 164 hips in the asymptomatic group. There was no correlation between FAI and body mass index or player position.

#### Ice hockey

FAI is a common cause of hip pain in professional ice hockey players [22, 65, 66]. Silvis *et al*. [20] obtained MRIs in 39 asymptomatic collegiate and professional hockey players. There was a 39% prevalence of alpha angles >55°. In a different study of 77 elite male ice hockey players (mean age 16.5 years; range 9–36 years), Siebenrock *et al.* [21] found that alpha angles were higher in athletes with closed physes compared with open physes (59° versus 50°). After physeal closure, 56% of hips had an abnormal alpha angle (>55°) compared with 6% of hips with an open physis. The authors concluded that ice hockey at an elite level during childhood is associated with an increased risk for cam-type deformity identified after physeal closure.

Philippon *et al.* [22] compared hip MRIs of 61 asymptomatic youth ice hockey players with 27 youth skiers as controls (age 10–18 years). The ice hockey players had significantly higher alpha angles than the control group. Additionally, the ice hockey players had a significant correlation between increased age and increased alpha angles, which was not present in the control group. The ice hockey group was 4.5 times more likely of having an alpha angle >55° (75%) than the skier group (42%).

#### Basketball

Siebenrock *et al.* [17] retrospectively compared 72 hips in elite male basketball players (mean age 17.6 years) with 76 asymptomatic hips in an age-matched control group. The mean hip internal rotation was 18.9° in the athletes compared with 30.1° in controls. While internal rotation was similar for both athletes and controls in the youngest group, athletes had a larger decrease in internal rotation than the controls in the older group. After comparing the youngest (age 9–12 years) with the oldest (age 22–26 years) groups, internal rotation decreased by an average 22.5° in athletes compared with only 10.2° in controls. Overall, the athletes had a 10-fold increased likelihood of having an alpha angle >55° in at least at one measurement position (89% compared with 9%).

Siebenrock *et al.* [23] studied the same cohort of elite basketball players and found that epiphyseal extension was increased in all positions in athletes compared with the control group. There was a significant increase in epiphyseal extension in athletes with open physes compared with controls with open physes. After physeal closure, this difference was still present, but to a lesser extent. They found a correlation between an alpha angle >55° and greater epiphyseal extension in the anterosuperior femoral head quadrant. The findings support the theory that the development of a cam-type deformity in athletes is related to an alteration of the growth plate rather than reactive bone formation.

#### Soccer

Gerhardt *et al.* [18] retrospectively studied the radiographs of 95 elite soccer players and found that 72% of male and 50% of female players had evidence of either a cam deformity and/or pincer lesion. Cam deformities were present in 68% of men (76.5% bilateral involvement) and 50% of women (90% bilateral involvement). The average alpha angle of all male players was 65.6°. Pincer lesions were found in 26.7% of men and 10% of women.

Johnson *et al.* [14] reviewed radiographs of 50 individuals who participated in high-level soccer during skeletal immaturity and 50 controls (age 18–30 years). The athlete group participated in youth soccer at least 3 days per week for at least 36 weeks per year between the ages of 10 and 14 years in men and 8–12 years in women. The control group did not participate in any sport beyond a recreational level and <2 days a week for <26 weeks per year during the same ages. In male athletes, 60% had an alpha angle >55° compared with 56% of controls. In female athletes, there was an abnormal alpha angle in 36% compared with 32% in controls. The controls in this study had a significantly higher incidence of cam deformities than in most other published reports [17, 56, 58–63]. The authors relied on the volunteers to recall the level of weekly sports participation, which may have led to recall bias.

Agricola *et al*. [13] compared the radiographs of 89 elite pre-professional soccer players (age 12–19 years) with controls. Soccer players had a higher prevalence of abnormal alpha angle (>60°) than controls (26% versus 17%). There was an increased prevalence of anterolateral flattening (56% versus 18%, *P* = 0.0001) and prominence (13% versus 0%, *P* < 0.03) in the soccer players compared with controls. The cam-type deformities were present early in adolescence and seemed to be more prevalent in soccer players. There was no widening or irregularity of the physis in any radiograph. Therefore, the authors concluded that a subclinical slipped capital femoral epiphysis (SCFE) is not the cause of most cam-type deformities.

In a follow-up study, Agricola *et al*. [27] prospectively followed 63 of the 89 pre-professional soccer players (mean age 14.43 years; range, 12–19 years) for a mean follow-up of 2.4 years. In hips with an open physis at baseline, the prevalence of cam deformities increased from 2.1% to 17.7% (*P* = 0.002). In those hips, the anterosuperior head–neck junction gradually changed from a concave shape (age 12 years) to being flattened (age 14 years) and eventually a convex shape (age 16 years). When studying the soccer players who were age 12 or 13 years at baseline, 84.1% had a normal appearance of the head–neck junction initially, which decreased to only 43.2% at follow-up (*P* < 0.001). After physeal closure, there was no significant increase in the prevalence or severity of cam deformity. The investigators also found that a decreased neck shaft angle (129.1° versus 133.6°; *P* = 0.001) and an increased epiphyseal extension (1.54 versus 1.43; *P* = 0.001) were associated with the presence of a cam deformity (alpha angle >60°). These data support the hypothesis that cam lesions are developed during a critical period of adolescence and that new lesions are not formed after physeal closure.

### Etiology of cam-deformity in athletes

There have been several hypotheses regarding the etiology of cam-deformity development in high-level adolescent athletes. One explanation is that high stresses lead to reactive bone formation at a location independent of the physis [67–70]. It has also been postulated that this injury is a subclinical SCFE that is asymptomatic during adolescence, but causes a cam deformity leading to FAI as an adult [24–26, 58, 71]. However, several authors have challenged this silent SCFE hypothesis [13, 57, 72, 73]. Beaule *et al.* [72] investigated the relationship between the alpha angle and beta angle (calculated with a similar method to alpha angle, but measuring the angle of the posterior head–neck junction). The basis of Beaule’s study was that if there is a silent SCFE, any increase in alpha angle would have a corresponding decrease in beta angle. The mean beta angle was significantly smaller in the symptomatic group compared with the control group. However, there was no significant relationship between an increasing alpha angle and decreasing beta angle. The authors concluded that although these findings do not exclude the possibility of a silent disruption of the proximal femoral epiphysis as a cause for head–neck abnormality in some individuals, other mechanisms may be more prevalent.

Siebenrock and Schwab [73] reported that a cam-type deformity in the absence of any previous hip pathology has distinctly different morphologic features than a residual or silent SCFE. In the post-SCFE hips, the femoral head center typically migrates posteriorly on lateral radiographs. However, this migration is not frequently observed in idiopathic cam-type deformities. The authors also discussed the posterior sloping angle (PSA), which evaluates the position of the epiphysis on the femoral neck and is 5° to 7° in normal hips compared with >12 in post-SCFE hips. Their institutional data of post-physeal closure basketball players with cam-type deformities showed a PSA of 4.1° ± 7.1° compared with age-matched controls who had a PSA of 6.7° ± 4.7°. Thus, they also concluded that the majority of cam-type deformities are not the sequelae of a silent SCFE.

As the result of the articles discussed above, the currently most accepted theory is that repetitive injury to the proximal femoral physis at a critical time period of development may result in the formation of a cam lesion. [17, 24–29] To evaluate this more scientifically, Carter *et al.* [24] performed a retrospective review of the MRI images of 17 adolescent patients (24 hips) with FAI. Linear mixed models were used to determine the association between the distance to the cam lesion and physeal status. The average alpha angles were 50.7°, 63.2°, 64.4° and 63.9° for the anterior, anterosuperior, superoanterior and superior radial MRI sections respectively. The average distance from the cam lesion to the physis was 7 mm. Patients with closed growth plates had a significantly greater distance between the cam lesion and physeal scar when compared with patients with more open growth plates. The findings suggest that the location of symptomatic cam-deformities in skeletally immature patients occurs at the level of the physis.

Some authors have compared this repetitive microtrauma of the hip to a similar process of the proximal humeral physis in Little Leaguer’s Shoulder and other growth plate disturbances in gymnasts [24, 74–76]. The stresses across the developing proximal femoral physis generated from running, kicking, ice-skating and jumping may be comparable with stresses across the proximal humeral physis in baseball pitching. Crockett *et al.* [77] found that the throwing shoulder in professional pitchers had increased humeral retroversion compared with both the player’s non-throwing shoulder and also non-throwing controls. By a similar mechanism, repetitive injury to the proximal femoral physis may lead to asymmetric growth and a decreased head–neck offset.

**Fig. 2.**
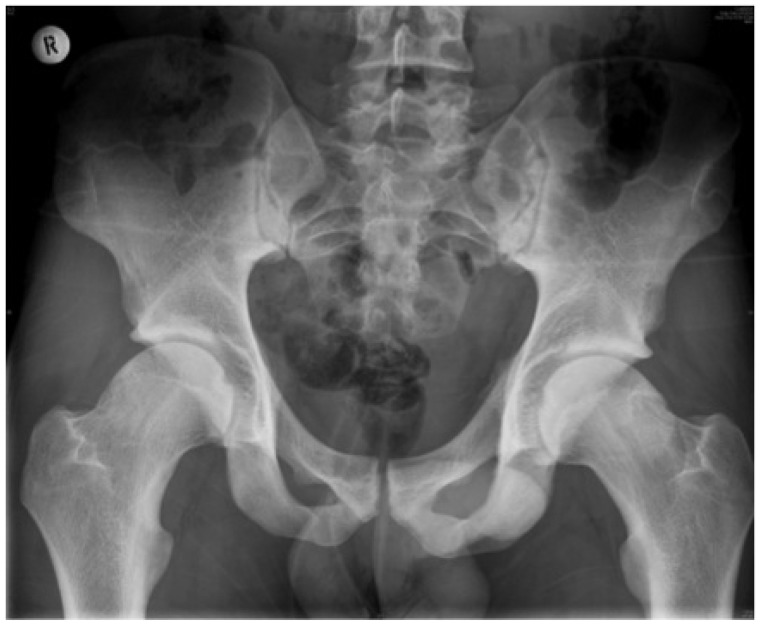
An AP Pelvis of an athlete with bilateral cam deformities. There is an increased epiphyseal extension of the lateral proximal femoral physis in both hips.

It is possible that increased physeal extension may represent an initial event preceding the development of a cam deformity (Fig. 2). In a study involving MR arthrography and the measurement of epiphyseal extension in patients with FAI (*n* = 15) and age- and gender-matched controls (*n* = 15), it was found in both groups that there was an inverse correlation between the amount of head–neck offset and the relative extension of the capital physeal scar in the cranial hemisphere of the femoral head [28]. Within the anterosuperior head quadrant of patients with cam deformities, there was a decrease in head–neck offset and an increase of the lateral epiphyseal extension compared with the controls. The findings of Jaramillo *et al.* [78] on a rabbit model of juxtaphyseal trauma provide physiological support to this concept. Metaphyseal injury resulted in interference with endochondral ossification, thickening of the growth plate and extension of cartilage into the metaphysis. They also found that juxtaphyeal trauma leads to the stimulation of endochondral ossification with thickening and extension of the epiphysis.

## CONCLUSION

Currently, there is no firm evidence that FAI is transmitted genetically. There is growing evidence that FAI, particularly cam-deformities, has a higher prevalence in athletes who performed at a high level during adolescence. There appears to be a critical period of time near physeal closure during which there is a high risk of development of a cam deformity. There are very few cases of FAI in patients younger than age 13 years and there is no increase in prevalence after physeal closure. However, the mechanism behind this pathology and the threshold duration of activity are not known. Further, there is a lack of research linking pincer impingement with athletic or other developmental stresses. A better understanding of FAI development may allow for preventative protocols to reduce the incidence of FAI and eventually hip osteoarthritis.

## CONFLICT OF INTEREST STATEMENT

None declared.
